# A rare case of Meigs syndrome in pregnancy

**DOI:** 10.11604/pamj.2019.33.36.18653

**Published:** 2019-05-16

**Authors:** Asaph Ziruma, Misai Hukuimwe, Michael Nyakura, Rumbidzai Majangara, Mervyn Venge

**Affiliations:** 1 Department of Obstetrics and Gynaecology, University of Zimbabwe, College of Health Sciences, Harare, Zimbabwe

**Keywords:** Meigs syndrome, fibrothecoma, pregnancy

## Abstract

We present a case of Meigs syndrome in a 19 year old woman. We suspected metastatic ovarian cancer after she had presented in her first pregnancy at 12 weeks gestation. Ultrasound scan had confirmed a complex solid mass in the left adnexa, measuring 7cm x 8cm, a viable 12 weeks pregnancy and gross ascites. She had elevated Ca 125 and serum beta - HCG. She went on to have a spontaneous miscarriage while being worked up for exploratory laparotomy. At laparotomy, a left sided solid ovarian mass 8cm x 10cm with a smooth surface and intact capsule was found. This was later confirmed to be a fibrothecoma at histology. The patient went on to recover without any further reaccumulation of ascites.

## Introduction

Meigs syndrome is the triad of benign ovarian tumor, ascites and pleural effusion that resolves after resection of the tumor. It is a rare diagnosis that can only be made after excluding ovarian cancer, which it closely mimics [1]. Histologically, the tumor may be a fibroma, thecoma, cystadenoma or granulosa cell tumor but fibroma constitutes the majority of the cases. Because the transdiaphragmatic lymphatic channels are larger in diameter on the right, the pleural effusion is usually right sided. The prognosis following excision of the tumor is good and similar to that of benign tumors. In 1%, there may be malignant transformation to fibrosarcoma. In a population-based hospital registry, ovarian cancer was the fifth most common cancer diagnosed during pregnancy [2]. Before widespread use of obstetric ultrasound, most adnexal masses in pregnant women remained unrecognized until cesarean delivery or until they became symptomatic, usually after delivery [3]. Adnexal masses in pregnancy may present with nonspecific symptoms that often mimic normal pregnancy [4]. Suspicion is often raised when the symptoms become exaggerated or severe enough to warrant investigation with an ultrasound scan. They may also present as a palpable mass during routine obstetric examination or with acute abdominal pain due to ovarian accident. Lastly, adnexal masses in pregnancy may also present with elevated maternal analytes. Alpha-fetoprotein and inhibin A are often used to screen for neural tube defects or trisomy 21 and unexplained elevation may be the first sign of an adnexal mass. Meigs syndrome is a rare condition that often mimics ovarian cancer and its diagnosis is only made after surgery and histological analysis.

## Patient and observation

We present a case of a 19 year old subsistence farmer. She presented in her first pregnancy at 12 weeks gestational age. She had been well until 3 months prior to presentation when she developed progressively worsening abdominal distension. The abdominal distention was not in keeping with the gestational age and was associated with vomiting bilious material. There were no associated diarrhea, constipation, urinary symptoms, vaginal discharge or bleeding. She was initially managed at a district hospital before she was referred to a provincial hospital. She decided not to go to the provincial hospital only to present 2 months later to our tertiary hospital casualty with worsening abdominal distention and severe shortness of breath. She had abdominal paracentesis done that produced 4 litres of straw coloured fluid. A right sided pleural tap of 200mls clear fluid was done after confirming pleural effusion on chest Xray. This temporarily relieved her respiratory distress. She had a microcytic anaemia, with haemoglobin of 6.5 g/dl. She was transfused 3 units of packed red blood cells. Her kidney function was normal. An echocardiography was ordered but was not done because of lack of funds. An ultrasound scan done after 4 days showed a normal singleton pregnancy at 12weeks 3 days gestation. Also noted was a complex solid mass in the left adnexa, 7cm x 8cm and gross ascites and pleural effusion. The rest of the abdomen was normal. Serum B HCG was 237000IU/L and Ca-125 was 125U/ml. She had a spontaneous miscarriage while being worked up for laparotomy. At laparotomy 12 litres of straw coloured fluid were drained and 20mls sent for cytology. There was a left sided solid ovarian mass approximately 8cm x 10cm in size with a smooth surface and intact capsule. The right ovary and fallopian tubes were normal. There were no lesions noted on the liver subdiaphragmatic spaces or paracolic gutters. The omentum was grossly normal and there were no palpable lymph nodes. Left salpingectomy and oophorectomy were done and specimens were sent for frozen section. The frozen section report showed a fibrothecoma. Cytological examination of peritoneal and pleural fluid samples showed absence of malignant cells. Biochemistry and microbiology of the fluids were not done due to lack of funds. Patient recovered very well post operatively and was discharged 3 days later. Follow up for 3 months showed the patient had not reaccumulated ascites and pleural effusion and she had recovered well from the operation. The Ca-125 had gone down to 37U/ml.

## Discussion

We have presented a case of a 19 year old woman who had a symptomatic complex adnexal mass at 12 weeks gestation, ascites and a pleural effusion who went on to have a spontaneous miscarriage while being prepared for surgery. Among other differentials, the possibility of a ruptured ectopic pregnancy was considered, with the low haemoglobin, an adnexal mass and fluid in the abdomen. However it was quickly ruled out because the patient was haemodynamically stable which was not in keeping with significant haemoperitomeum. She was managed as a patient with probable metastatic ovarian cancer. Adnexal masses in pregnancy have an incidence of 0.1 to 2.4 percent. While the majority of these tumors are benign, the possibility of ovarian cancer should always be considered [5]. Widespread use of ultrasound scan has significantly improved the diagnosis of adnexal masses in pregnancy. Historically it was common for these masses to remain unrecognized until cesarean delivery or until they became symptomatic, usually after delivery [3]. As we saw in our case, adnexal masses in pregnancy may present with nonspecific symptoms that may appear like exaggerated symptoms of pregnancy. The incidence of Meigs syndrome is 1% of all fibromas of the ovaries, ascites occurs in 10-15% of fibromas when the tumor size is more than 10 cm and hydrothorax is found in 1% [6]. The pathophysiology of ascites in Meigs syndrome is poorly understood. It has been suggested that irritation of the peritoneal surfaces by a solid ovarian tumor could stimulate the production of ascites. It has also been found that only tumors larger than 10cm in diameter with a myxoid component to the stroma are associated with ascites [7]. Other possible mechanisms are direct pressure on surrounding lymphatics or vessels, hormonal stimulation and tumor torsion. It may be due to release of inflammatory mediators from the tumor, leading to increased capillary permeability. It has been theorized that pleural effusion is a result of transfer of ascitic fluid via transdiaphragmatic lymphatic channels. However the size of the pleural effusion is largely independent of the amount of ascites and it may be left sided or bilateral [1]. Pseudo-Meigs syndrome consists of pleural effusion and benign tumors of the ovary other than fibromas. These benign tumors include those of the fallopian tube or uterus and mature teratomas, strumaovarii and ovarian leiomyomas. Atypical Meigs is characterized by a benign pelvic mass with right-sided pleural effusion but without ascites. As in Meigs syndrome, pleural effusion resolves after removal of the pelvic mass. Surgery is necessary to confirm the diagnosis. In pregnancy, surgical resection of asymptomatic masses should be done after the first trimester if the mass is still present. Adnexal masses more than 10cm, wholly solid or containing solid, cystic areas or papillary areas and septae are associated with high risk of malignant potential [5, 8]. Adnexal masses that do not meet this criteria are more likely to be benign, physiological and more likely to resolve during the pregnancy [5]. Expectant management is also appropriate if the sonographer is reasonably certain that the tumor is benign. After the first trimester, most functional cysts have already resolved.

Organogenesis is mostly complete, minimizing the risk of drug induced teratogenesis. The placenta has taken over hormone production from the corpus luteum thereby minimizing the risk of pregnancy loss due to possible reduction in progesterone levels after tumor resection. Spontaneous pregnancy loss due to fetal abnormalities is likely to have occurred and will not likely be attributed to surgery done after the first trimester. If the ovarian mass persists throughout pregnancy, the risk of malignancy is up to 10% [8, 9]. A significant portion of these are germ cell and epithelial tumors with low malignancy potential and favourable prognosis. Preoperative evaluation of adnexal masses in pregnancy is usually limited to ultrasound imaging. Where ultrasound scan cannot adequately evaluate the pathology, magnetic resonance imaging (MRI) should be used if available. The precision of MRI may make it possible to opt for expectant management until delivery [10]. In pregnancy, routine chest radiography using X-ray is not advised, except if history and examination suggest pulmonary disease. In those cases, the abdomen and pelvis should be shielded. Computed tomography is avoided if ultrasound and MRI can provide the required information [11, 12]. Tumor markers are not routinely recommended for adnexal tumors in pregnancy. Tumor markers are usually recommended only after confirmation of malignancy. Pregnancy associated adnexal tumors are usually benign. Tumor markers used in nonpregnant women are difficult to interpret in pregnant women. Human chorionic gonadotropin, carcinoembryonic antigen and cancer antigen 125 are involved in biological functions associated with fetal development, differentiation and maturation. They are normally elevated and fluctuating during normal pregnancy [13]. However, after 15 weeks of gestation, CA 125 may be useful as a tumor marker because its elevation is less likely to be part of normal pregnancy. Some authors suggest a maternal serum alpha fetoprotein level above 9 multiples of the median should prompt concern for possible germ cell tumor [14]. Serum lactate dehydrogenase (LDH) is not elevated in normal pregnancy. It is elevated with ovarian dysgerminoma and is reliable as a tumor marker in pregnant women. Serum inhibin A is a useful tumor marker for ovarian granulosa cell tumors in non pregnant women. However, because it is made by the placenta, it cannot be reliably used in pregnancy. Just like inhibin A, HCG is produced in large quantities during pregnancy, limiting its usefulness as a tumor marker. Human epididymis protein 4 (HE4) is unaffected by pregnancy and therefore may be helpful in evaluating pelvic masses in pregnancy [15].

## Conclusion

Most benign tumors in pregnancy are benign and they usually resolve spontaneously during the pregnancy. However all pelvic tumors should be evaluated clinically for malignancy potential. If there is significant risk of malignancy, the patient should have surgery as soon as possible, preferably after the first trimester.

## Competing interests

The authors declare no competing interests.
